# CHD7 promotes proliferation of neural stem cells mediated by MIF

**DOI:** 10.1186/s13041-016-0275-6

**Published:** 2016-12-13

**Authors:** Shigeki Ohta, Tomonori Yaguchi, Hironobu Okuno, Hervé Chneiweiss, Yutaka Kawakami, Hideyuki Okano

**Affiliations:** 1 Department of Physiology, Keio University School of Medicine, 35 Shinanomachi, Shinjuku-ku, Tokyo, 160-8582 Japan; 2 Division of Cellular Signaling, Institute for Advanced Medical Research, Keio University School of Medicine, 35 Shinanomachi, Shinjuku-ku, Tokyo, 160-8582 Japan; 3 Sorbonne Universités, UPMC Univ Paris 06, INSERM, CNRS, Neurosciences Paris Seine - Institut de Biologie Paris Seine (NPS - IBPS), 75005 Paris, France; 4 Present Address: Division of Cellular Signaling, Institute for Advanced Medical Research, Keio University School of Medicine, 35 Shinanomachi, Shinjuku-ku, Tokyo, 160-8582 Japan

**Keywords:** CHD7, MIF, Neural stem/progenitor cells, Glioma initiating cells

## Abstract

Macrophage migration inhibitory factor (MIF) plays an important role in supporting the proliferation and/or survival of murine neural stem/progenitor cells (NSPCs); however, the downstream effectors of this factor remain unknown. Here, we show that MIF increases the expression of Pax6 and Chd7 in NSPCs in vitro. During neural development, the chromatin remodeling factor Chd7 (chromatin helicase-DNA-binding protein 7) is expressed in the ventricular zone of the telencephalon of mouse brain at embryonic day 14.5, as well as in cultured NSPCs. Retroviral overexpression of Pax6 in NSPCs increased Chd7 gene expression. Lentivirally-expressed Chd7 shRNA suppressed cell proliferation and neurosphere formation, and inhibited neurogenesis in vitro, while decreasing gene expression of Hes5 and N-myc. In addition, CHD7 overexpression increased cell proliferation in human embryonic stem cell-derived NSPCs (ES-NSPCs). In Chd7 mutant fetal mouse brains, there were fewer intermediate progenitor cells (IPCs) compared to wildtype littermates, indicating that Chd7 contributes to neurogenesis in the early developmental mouse brain. Furthermore, *in silico* database analysis showed that, among members of the CHD family, CHD7 is highly expressed in human gliomas. Interestingly, high levels of CHD7 gene expression in human glioma initiating cells (GICs) compared to normal astrocytes were revealed and gene silencing of CHD7 decreased GIC proliferation. Collectively, our data demonstrate that CHD7 is an important factor in the proliferation and stemness maintenance of NSPCs, and CHD7 is a promising therapeutic target for the treatment of gliomas.

**Electronic supplementary material:**

The online version of this article (doi:10.1186/s13041-016-0275-6) contains supplementary material, which is available to authorized users.

## Introduction

Macrophage migration inhibitory factor (MIF) is known to be a proinflammatory factor in many diseases, including atherosclerosis and rheumatoid arthritis [1]. Additionally, MIF has been shown to induce cell proliferation in immune cells and prostate cancer cells [2–4]. Studies of MIF function in animals suggest that this factor may play additional unknown roles in other diseases. In the central nervous system (CNS), MIF expression has been reported in the rat forebrain ventricular zone [5], yet the function of MIF in the CNS and in NSPCs had not yet been clarified [6]. We previously reported that MIF supports the proliferation and/or survival of murine NSPCs in vitro [7]. We have also identified Sox6 as a MIF downstream signaling molecule in mouse NSPCs [8]. We also recently reported that MIF supports the proliferation of GICs through TP53 regulation [9].

In the present study, we sought to clarify the function of CHD7 (chromatin helicase-DNA-binding protein seven), which has a chromodomain, a helicase domain, a SANT-like (switching-defective protein three (Swi3), adaptor 2 (Ada2), nuclear receptor co-repressor (N-CoR), transcription factor (TF) IIIB-like) domain, and a BRK (Brahma and Kismet) domain [10] in mouse NSPCs and human ES-NSPCs. CHD7 is a member of the CHD family, which regulates chromatin remodeling and gene regulation through direct DNA-binding in a manner dependent on the biological context, although the detailed mechanisms underlying this activity remain unknown [11, 12]. In human neural crest cells and mouse ES cells, CHD7 targets active gene enhancers regulating cell-specific gene expression [13, 14]. Mutations in CHD7 are known to be closely linked to CHARGE syndrome (Coloboma, Heart defects, Atresia of the choanae, Retardation of growth and development, Genital hypoplasia, and Ear abnormalities, including deafness and vestibular disorders), also known as multiple congenital abnormality [10]. In human, the mutation of CHD7 was also identified in an autism spectrum disorder proband [15]. In mouse, it has been reported that Chd7 regulates the neural differentiation of hippocampal NSPCs through SoxC or Hes5 [16, 17]. It has also been reported that Chd7, in cooperation with Sox10, regulates the onset of oligodendrocyte differentiation and myelination, as well as remyelination after demyelinating injury [18]. Physiological interaction between CHD7 and SOX2 has also been reported in human NSPCs [19]. CHD7 may thus play multiple biological roles in concert with specific co-activators depending on spatial and developmental status [20]. However, it remains unclear how CHD7 is regulated by upstream regulators, especially in NSPCs.

In the present study, we show that Chd7 is expressed in the ventricular zone and subventricular zone (SVZ) of the ganglionic eminence (GE) and cortex in the mouse developmental brain, in which NSPCs are located. We further show that *Chd7* expression is increased by MIF in NSPCs in vitro, and that this effect is mediated by the transcription factor *Pax6*. In addition, based on the same mechanism, we find that CHD7 supports the proliferation of human ES- NSPCs, as well as the cell proliferation of GICs, suggesting that this molecule may represent a new therapeutic target in glioma.

## Methods

### Animals

All interventions and animal care procedures were performed in accordance with the Laboratory Animal Welfare Act, the Guide for the Care and Use of Laboratory Animals (National Institutes of Health, USA), and the Guidelines and Policies for Animal Surgery provided by the Animal Study Committee of the Keio University and were approved by the Animal Study Committee of Keio University (IRB approval number 12017-0). Pregnant C57BL/6 J mice were purchased from Sankyo Labo Service (Tokyo, Japan). Chd7 mutant mice (Chd7^Whi^, EM;04923) were purchased from the European mouse mutant archive (www.infrafrontier.eu) and maintained on a C3HeB/FeJ background. Genotyping was performed following to the previous report [21].

### Cell culture

NSPCs were isolated from mouse E14.5 GEs, and the cells were cultured as neurospheres [22] at a cell density of 50 cells/μl in neurosphere culture medium (NSP medium) consisting of neurobasal medium (Thermo Fisher Scientific, Carlsbad, CA, www.thermofisher.com) supplemented with B27 (Thermo Fisher Scientific), human recombinant (hr) EGF (20 ng/ml; Peprotech, Rocky Hill, NJ, www.peprotech.com), and hrFGF2 (10 ng/ml; Peprotech). Neurosphere formation assays were performed at low density (20 cell/μl) in a 96-well plate. In the experiments using NSPCs, recombinant mouse MIF (R&D systems, Minneapolis, MN, www.rndsystems.com) and MIF inhibitor ISO-1 (Calbiochem, La Jolla, CA, www.merckmillipore.com) were used. NSPCs derived from human ESCs (Human ES Cells H9-Derived) were purchased from Thermo Fisher Scientific, and then cultured and differentiated according to the product manual. Human GICs were obtained as described previously [23]. Human astrocytes, U87MG, U251 and NSPCs were cultured as reported by Fukaya et al., [9]. The study using human NSPCs was carried out in accordance with the principles of the Helsinki Declaration, and the Japan Society of Obstetrics and Gynecology. Approval to use human fetal neural tissues was obtained from the ethical committees of both Osaka National Hospital and Keio University. Written informed consent was obtained from all parents through routine legal terminations performed at Osaka National Hospital.

### RNA extraction and quantitative (q) RT-PCR

Total RNA was isolated from tissues or cultured cells using TRIZOL (Thermo Fisher Scientific). Total RNA (0.5 μg) was subjected to the cDNA Synthesis using ReverTra Ace® qPCR RT Master Mix with gDNA Remover (Toyobo, Osaka, Japan, http://lifescience.toyobo.co.jp). Quantitative RT-PCR analysis was performed with a FastStart Universal SYBR Green Master (Roche, Tokyo, https://roche-biochem.jp) or THUNDERBIRD Probe qPCR Mix (Toyobo), using the ABI prism 7900 HT Sequence Detection System (Applied Biosystems, Life Technologies, Carlsbad, www.appliedbiosystems.com). The PCR conditions were as follows: one cycle of 5 min at 95 °C, followed by 40 cycles of 95 °C for 30 s, 60 °C for 60 s, and 72 °C for 30 s (SYBR), one cycle of 1 min at 95 °C, followed by 40 cycles of 95 °C for 30 s, 60 °C for 60 s (Taqmanprobe). PCR reactions were performed in triplicate. Relative gene expression levels were determined using the ΔΔ*C*t-method. GAPDH mRNA levels were used as the internal normalization control. The primer sequences are listed in Additional file 1: Table S1 and described in the previous study [7, 8] and the designs of the NSE, GFAP, and CNPase primers were made following the directions of the ATRC Reagent Bank (http://neurodiscovery.harvard.edu/atrc-reagent-bank).

### Retrovirus and lentivirus production

Human full-length CHD7 cDNA (pF1KE9669 Flexi ORF Clone, PROMEGA, www. promega.jp) was subcloned into the pF5K CMV-neo Flexi vector vector (Promega), and a pMX-Pax6 plasmid (mouse) was obtained from Addgene (Cambridge, MA, www.addgene.org). Human PAX6 expression vector (pCS-PAX6, BC011953) was purchased from Transomic (Huntsville, AL, www.transomic.com). Recombinant lentiviruses were produced by the shCHD7 lentivirus vector (EHS4430-98514866, EHS4430-98714285, Open Biosystems. dharmacon.gelifesciences. com) or control shRNA vector (RHS4346, Open Biosystems). Retrovirus and lentivirus production including shMIF were performed as described previously [7, 8].

### Western blot analysis

Cell lysates were prepared using RIPA buffer (25 mM Tris–HCl, 150 mM NaCl, 1% NP-40, 1% sodium deoxycholate, and 0.1% SDS, pH 7.6) containing protease inhibitors (Cocktail Tablet; Roche). Lysates were centrifuged at 14,000 × *g* for 15 min at 4 °C, and the protein concentration of each sample was determined using a Bio-Rad protein assay kit (Bio-Rad, Tokyo, Japan, www.bio-rad.com) with bovine serum albumin as a standard. Identical amounts of protein were electrophoresed in 10% SDS-PAGE gels and transferred to a nitrocellulose membrane. Blots were blocked with Blocking One™ (Nacalai Tesque, Kyoto, Japan, www.nacalai.co.jp) at RT for 1 h, then incubated with primary antibodies overnight at 4 °C as follows: CHD7 (1:100; BETHYL Laboratories, Montgomery, TX,www.bethyl.com), p21(1:1000; MBL, ruo.mbl.co.jp), p27 (1:1000; Cell signaling Technology), N-MYC (1:100; Abcam, www. Abcam.co.jp), Lamin-B1(1:1000; Abcam), and actin (1:5000; Sigma, www.sigmaaldrich.com). After three washes in TBST (20 mM Tris–HCl, 150 mM NaCl, and 0.02% Tween-20, pH 7.4), the blots were incubated with the appropriate secondary antibodies conjugated with horseradish peroxidase (1:4000, anti-rabbit and anti-mouse; GE Healthcare, Tokyo, Japan, http://www.gelifesciences.co.jp) for 1 h at room temperature. Signals were detected with ECL-Plus Substrate (GE Healthcare) and exposed to Hyperfilm (GE Healthcare).

### Cell proliferation and apoptosis assay

Cell viability was assessed using Cell Titer-Glo Luminescent Cell Viability Assay kits (Promega) and a luminometer (EnVision™ multilabel reader, Perkin Elmer, Waltham, MA, www.perkinelmer.com). Single cells dissociated from neurospheres were seeded onto 96-well plates at a density of 5x10^3^ cells/well and activity was assayed on the days described.

### Immunocytochemistry and immunohistochemistry

Mice embryonic brains were removed and fixed in 4% paraformaldehyde (PFA) in 0.1 M phosphate-buffered saline (PBS), cryoprotected in 30% sucrose solution in PBS, and embedded in O.C.T. compound (Sakura Finetek, Tokyo, Japan, www.sakura-finetek.com). Adult mice were killed by anesthetic overdose and perfused transcardially with 4% PFA in PBS, pH 7.2. Brains were postfixed in the perfusion solution overnight at 4 °C, then cryoprotected for at least 24 h in 30% sucrose in PBS and embedded as above. Brain blocks were sectioned in the appropriate plane in 14 μm slices. After blocking with 10% goat normal serum in 0.1 M PBS, brain slices were incubated in 5% goat normal serum in 0.1 M PBS + 0.3% Triton X-100 with the following primary antibodies: rabbit anti-CHD7 (1:100; BETHYL laboratories), rabbit anti-Tbr2 (1:500; Abcam, Cambridge, MA, www.abcam.com), pH3 (1:1000; Abcam), Ki67 (1:1000; Abcam), anti-Nestin (1:100; Abcam), Pax6 (1:200; MBL), mouse anti-NeuN (1:100; Millipore). Application of the primary antibodies was followed by incubation of the brain slices with secondary antibodies labeled with Alexa Fluor 488, and 568 (1:400; Thermo Fisher Scientific). For immunocytochemical studies, cells were fixed with PBS containing 4% PFA for 20 min at room temperature, and the cells were subjected to immunofluorescence staining using the following primary antibodies: rabbit anti-CHD7 (1:100; BETHYL laboratories), mouse anti-β-tubulin type III (TuJ1) (1:1000; Sigma), mouse anti-MAP2 (1:200; Sigma), mouse anti-NeuN (1:100; Millipore), mouse anti-CNPase (1:250; Sigma), mouse anti-GFAP (1:400; Sigma) and rabbit anti-GFAP (1:400; Biomedical Technologies, Stoughton, MA, https://www.alfa.com). After PBS washes, antibody binding was visualized using either Alexa Fluor 488 or 568-conjugated secondary antibodies (Thermo Fisher Scientific), and the nuclei were stained with DAPI (4’,6-diamidino-2-phenylindole, Thermo Fisher Scientific). In mouse NSPCs differentiation assays, single dissociated cells of cultured neurospheres were plated on poly-L-lysine coated glass slips at a density of 2x10^5^ cells/cm^2^ in NSP medium without growth factors for 4 days, and then subjected to qPCR analysis. In mouse NSPCs differentiation assays were performed according to the previous study (7). In human NSPCs differentiation assays were performed according to the product protocol. In the immunocytochemical analyses, neurons and astrocytes were analyzed 2 weeks and 3 weeks, respectively, after differentiation, and at least 10 different viewing fields were counted using LMS700 confocal microscopy (Zeiss, Tokyo, Japan, www.zeiss.co.jp).

### Statistical analysis

All values are expressed as mean ± S.D or S.E. Student’s *t* tests were used to determine the statistical significance of differences between groups (*P < 0.05, **P < 0.01).

## Results

### Chd7 is a downstream target of MIF in mouse NSPCs

**Fig. 1:**
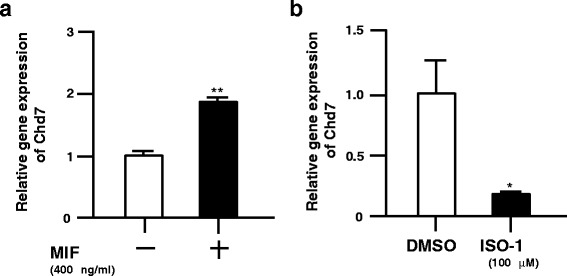
MIF regulates gene expression of Chd7 in mouse NSPCs. **a**, MIF treatment (400 ng/ml) for 24 h increases Chd7 gene expression. **b** MIF antagonist (ISO-1) treatment (100 μM) for 24 h decreased Chd7 gene expression in NSPCs. Data are derived from three independent experiments. Error bars indicate S.D. values; *P < 0.05, **P < 0.01 versus control; Student’s *t*-test

We first performed qRT-PCR analysis to examine whether Chd7 gene expression changes on MIF treatment of NSPCs. MIF treatment increased the RNA level of Chd7 in NSPCs (Fig. 1a). In contrast, cell treatment with ISO-1, a MIF antagonist, led to a decrease in Chd7 RNA levels in NSPCs (Fig. 1b). We have also observed that the CHD7 protein changes correlate closely with changes in its gene expression (data not shown). Together, these results suggest that the Chd7 gene is a downstream target of MIF signaling in NSPCs in vitro.

### Chd7 is expressed in mouse embryonic brain NSPCs

**Fig. 2:**
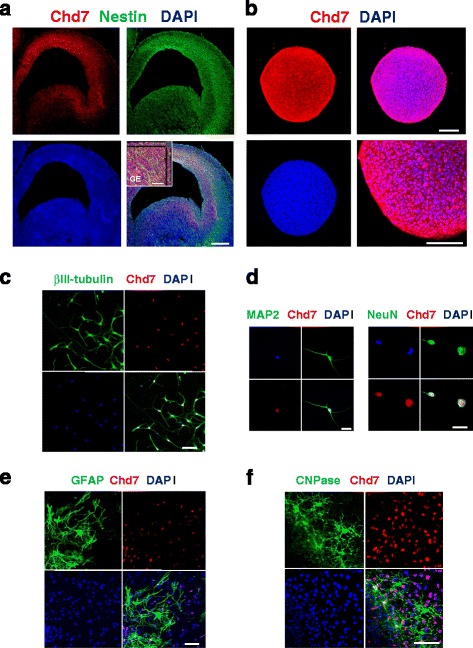
Chd7 expression in NSPCs. **a**, Immunohistochemistry of Chd7-positive cells (*red*) were co-labeled with nestin (*green*) in E14.5 mouse brain. **b**, Immunocytochemistry of neurospheres using Chd7 antibody. **c**–**f**, Chd7 expression in differentiated neural cells generated from embryonic day 14.5 brain GE-derived neurospheres 5 days after in vitro differentiation. Many Chd7-positive cells were observed in differentiated neurons identified by βIII-tubulin (**c**), MAP2 and NeuN (**d**), although a small number of Chd7-positive cells was observed in differentiated glial cell types identified by the following markers: GFAP (**e**), and CNPase (**f**). Scale bar; 200 μm (**a**), 100 μm (**b**), 50 μm (**c**, **d**, **e**), 20 μm (Enlarged image **a**, **d**)

Expression of the Chd7 protein in the mouse developmental brain at E14.5 was examined by immunofluorescence. Chd7 was expressed in the ventricular zone of the cortex and GE. The Chd7-positive cells also expressed nestin, a known marker of NPSCs (Fig. 2a). This expression pattern of Chd7 suggests that Chd7 is expressed in NSPCs in vivo. Next, Chd7 protein expression was examined in cultured NSPSs. Chd7 was expressed in neurospheres generated from E14.5 GEs (Fig. 2b). In addition, Chd7 expression was analyzed in the neural differentiated lineage in vitro, indicating that many Chd7-positive cells were observed in neurons, and some Chd7-positive cells were found in glial lineage (glia and oligodendrocyte) cells (Fig. 2c–f, Additional file 2: Figure S1C). However, western blot analysis showed a significant reduction of Chd7 protein in the differentiated cells compared to undifferentiated NSPCs, suggesting a functional role of Chd7 in NSPCs (Additional file 2: Figure S1D).

### Chd7 supports cell survival and the self-renewal ability of NSPCs

**Fig. 3:**
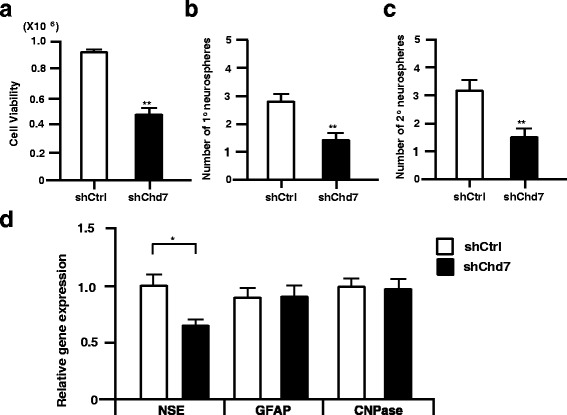
Chd7 regulates mouse NSPCs proliferation and self-renewal in vitro. **a**, Chd7 targeting using lentiviral shRNA significantly reduced NSPC growth compared to control shRNA, as assessed using a Cell Titer-Glo Assay Kit 4 days after infection. **b**, In the primary neurosphere formation assay, single dissociated cells of neurospheres generated from E14.5 brain were plated onto a 96-well plate and infected with lentiviruses encoding control shRNA or Chd7 shRNA. The cells were cultured in the presence of both EGF and FGF2 **c**, In the secondary neurosphere assay, neurospheres infected with a lentivirus encoding control shRNA or Chd7 shRNA were cultured in the presence of both EGF and FGF2 for 5 days. Then neurospheres were dissociated into single cells and seeded onto a 96-well plate at a cell density of 20 cells/μl in the presence of EGF and FGF2. **d**, Neurospheres infected with lentivirus expressing either control or Chd7 targeting shRNA were cultured in the presence of both EGF and FGF2 for 5 days and then dissociated and cultured onto the poly-D-lysine coated glass slip for 4DIV in the absence of growth factors. The gene expression of neural marker (NSE), an astrocyte marker (GFAP), or an oligodendrocyte marker (CNPase) was quantified by qRT-PCR. For the graphs, the data were compiled from three independent experiments. Error bars indicate S.E. values; **P < 0.01 versus control; Student’s *t*-test

To identify the function of Chd7 in NSPCs, we first examined changes in NSPC viability by lentivirus-mediated silencing of Chd7. Chd7 gene silencing decreased cell viability by half (0.51 ± 0.08 fold) 4 days after infection (Fig. 3a). The same effect was confirmed by a different Chd7-targeting lentivirus vector (Additional file 2: Figure S1). Moreover, we performed the neurosphere-forming assay using a lentivirus expressing Chd7-shRNA in NSPCs, Chd7 silencing by lentivirus expression of shRNA-Chd7 in NSPCs attenuated the formation of primary and secondary neurospheres by 0.50 ± 0.31 fold and 0.48 ± 0.24 fold, respectively (Fig. 3b, c). Taken together, Chd7 may support NSPCs cell survival and/or proliferation. Next, we tested changes in the multi-lineage differentiation potential of NSPCs upon Chd7 gene silencing. NSPCs infected with lentivirus expression of shRNA-Chd7 were cultured for 5 days, and then replated and cultured for four additional days in the absence of growth factors. Then, the gene expression level of differentiation into each neuronal cell type was assessed by qRT-PCR. In this assay, only the neuron marker gene (NSE, neuron-specific enolase) expression was reduced, although astrocytes (GFAP), and oligodendrocytes (CNPase) marker genes expression were not changed with a statistical value (Fig. 3d). The same defect in neuronal differentiation by Chd7 gene silencing was also observed on immunocytochemical analysis (Additional file 3: Figure S2). Furthermore, we examined whether Chd7 plays a role in cell proliferation in response to MIF in NSPCs. While MIF treatment of control cells resulted in an increase of cell viability 4 days after treatment (1.31 ± 0.14 fold compared to control), this effect was blunted by knockdown of Chd7 using lentivirus expressing Chd7-shRNA, further supporting that Chd7 is a downstream effector of MIF signaling in NSPCs (Additional file 3: Figure S2).

### Upstream and downstream signal of Chd7 in NSPCs

**Fig. 4:**
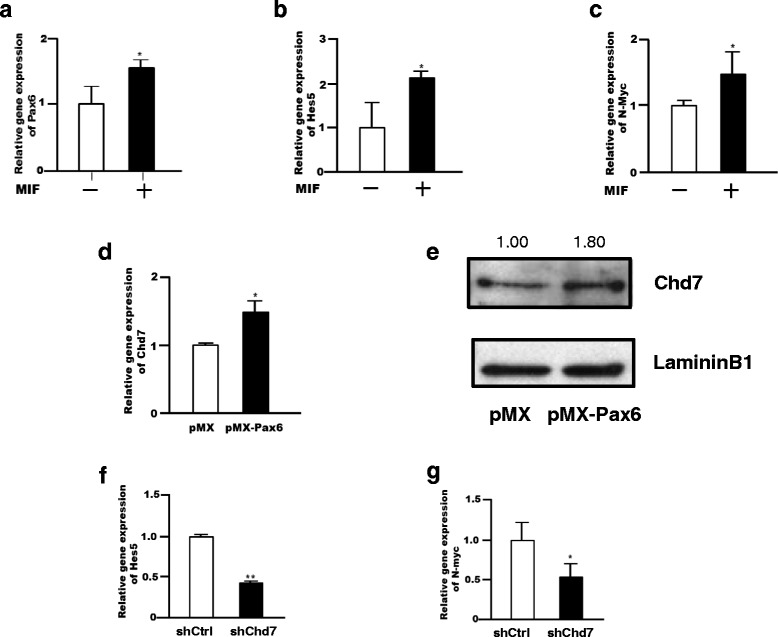
Analysis of MIF-Chd7 signaling cascade in mouse NSPCs. **a**–**c**, MIF increases the expression of Pax6 (**a**), Hes5 (**b**) and N-myc (**c**) in NSPCs 24 h after MIF treatment. Each mRNA expression level was normalized against GAPDH mRNA levels, then expressed relative to the normalized value of controls. **d**, **e**, Retroviral Pax6 overexpression in NSPCs led to an increase in Chd7 gene (**d**) and protein (**e**) 5 days after infection. **f**, **g**, Lentiviral CHD7-shRNA expression decreased the gene expression of Hes5 (**f**) and N-myc (**g**) in NSPCs 5 days after infection. For the graphs, the data were compiled from three independent experiments. Error bars indicate S.D. values; **P < 0.01 versus control; Student’s *t*-test

Chd7 was identified as a MIF downstream target in NSPCs (Fig. 1). In addition, N-myc, Hes5, and Pax6 gene expression were increased upon treatment of NSPCs with MIF (Fig. 4a–c). In human and murine, a Pax6 binding site locates in the upstream Chd7 promoter region of transcription starting site (http://www.sabiosciences.com/chipqpcrsearch.php). Thus, we examined whether Chd7 expression is regulated by Pax6 in NSPCs. Retroviral over-expression of Pax6 increased Chd7 gene expression (Fig. 4d) that resulted in an increased CHD7 protein expression (Fig. 4e), indicating Chd7 is a downstream target of MIF and Pax6 in NSPCs. Moreover, Hes5 and N-myc gene expression were decreased by Chd7 gene silencing using lentivirus expressing shChd7-shRNA in NSPCs, suggesting a cascade of independent events where MIF increases Pax6 expression which then increases Chd7 expression, resulting in an increase in N-myc and Hes5 gene expression (Fig. 4f, g).

### Chd7 regulates population of IPCs in mouse developmental neocortex

**Fig. 5:**
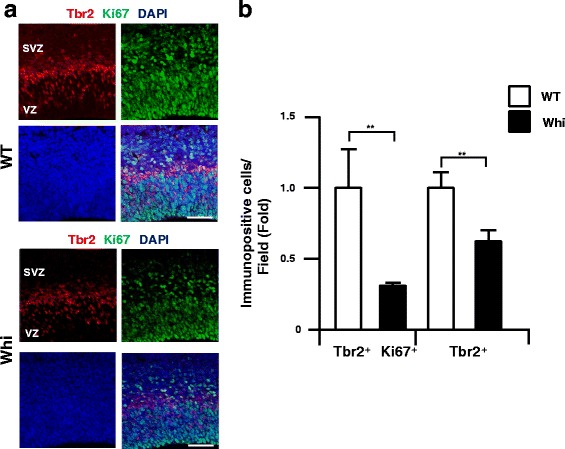
Decrease of Tbr2-positive cells in Chd7 mutant mouse in the developing cerebral cortex**. a**, The expression of Tbr2-positive IPCs cells (*red*) were co-labeled with Ki67 antibody (*green*) in Chd7 mutant (whi/+) and wild-type (WT) in E14.5 mouse embryonic brain. The nuclei were stained using DAPI (*blue*). **b**, The number of Tbr2/Ki67 and Tbr2-positive cells were decreased in Chd7 mutant (whi, *n* = 5) compared to wild-type (*n* = 6). Error bars indicate S.D. values; **P < 0.01 versus control; Student’s *t*-test. Scale bar; 20 μm

To analyze the role of Chd7 in the mouse developmental brain, whirligig heterozygous Chd7 mutant mouse (Chd7^Whi/+^) was analyzed at E14.5. Chd7^Whi/+^ carries the heterozygous mutation c.2918 G > A, generating a stop codon, having similar symptoms seen in CHARGE syndrome patients. In addition, the Chd7^Whi/Whi^ mouse dies shortly at E10.5 [24], thus we analyzed the Chd7^Whi/+^ mouse at E14.5. In the neocortex, the number of cortical IPCs (Tbr2^+^ cells) was decreased in the mutant mouse compared to the wild type littermates (0.61 ± 0.08 fold compared to control)(Fig. 5). In addition, the number of Tbr2/Ki67 double-positive cells was also decreased in the Chd7 mutant mouse compared to the wild type littermates (0.31 ± 0.01 fold compared to control), indicating a decrease of neuronal progenitors (Fig. 5). In addition, the number of Pax6/pH3-double positive cells (pH3, a marker for M-phase cells, [25]) was also smaller in the apical ventricular zone of E14.5. Chd7^Whi/+^ mouse compared to wild-type littermates (0.69 ± 0.17 fold compared to control), suggesting a reduction in some population of NSPCs caused by the decrease of CHD7 protein (Additional file 4: Figure S3A). In addition, a mature neural cell population (NeuN-positive cells) was also decreased in the Chd7^Whi/+^ mouse cortical plate compared to wild-type littermates (0.74 ± 0.08 fold compared to control), indicating a decrease in NSPCs and IPCs in the Chd7^Whi/+^ mouse (Additional file 4: Figure S3B).

### Chd7 supports cell survival and/or proliferation of human ES-NSPCs

**Fig. 6:**
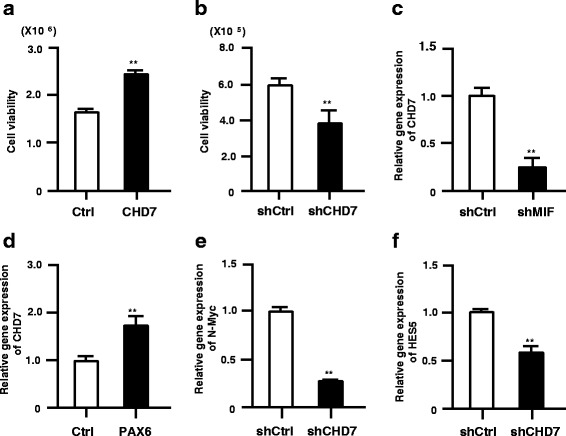
CHD7 signaling cascade in human ES-NSPCs. **a**, **b**, Over-expression of CHD7 increases cell proliferation in human ES-NSPCs 5 days after transfection (**a**). In contrast, CHD7 targeting using lentiviral shRNA significantly reduced human ES-NSPCs growth compared to control shRNA, as assessed using a Cell Titer-Glo Assay Kit 3 days after infection (**b**). **c**, Lentiviral MIF-shRNA expression led to a decrease of CHD7 gene expression 2 days after infection. **d**, PAX6 over-expression in human ES-NSPC led to an increase of CHD7 gene expression 2 days after transfection. **e**, **f**, Lentiviral CHD7-shRNA expression decreased the gene expression of N-MYC (**e**) and HES5 (**f**) in human ES-NSPC 2 days after infection. Data are representative of three independent experiments. Error bars indicate S.D. values; **P < 0.01 versus control; Student’s *t*-test

We also tested whether CHD7 can support the cell proliferation and or survival in human ES-NSPCs as seen in mouse NSPCs. We used human ES-NSPCs, which were cultured as attachment cells. The differentiation potential of the human ES-NSPCs into neuron and glia was confirmed and cell viability of human ES-NSCs was also increased by recombinant human MIF treatment (data not shown). Transient CHD7 gene over-expression in human ES-NSPCs increased in the cell proliferation (1.49 ± 0.04 fold compared to control) (Fig. 6a); in contrast, human ES-NSPCs infected with lentivirus expression of shRNA-CHD7 decreased cell proliferation (0.31 ± 0.01 fold compared to control) (Fig. 6b), which is consistent with our experimental results in mouse. Moreover, lentiviral MIF gene silencing decreased CHD7 gene expression in human ES-NSPCs similar to mouse NSPCs (Fig. 6c). Furthermore, as seen in mouse NSPCs, transient over-expression of human PAX6 up-regulated the CHD7 gene expression in human ES-NSPCs (Fig. 6d). In human ES-NSC, gene silencing of CHD7 also downregulated N-MYC and HES5 gene expression as seen in mouse NSPCs (Fig. 6e, f). Similar to the case in the mouse system, MIF silencing in human ES-NSPCs downregulated the gene expression of PAX6, N-MYC, and HES5 (Additional file 5: Figure S4). In addition, the change of neuronal differential potential by CHD7 gene silencing was confirmed by immunocytochemical analysis, and the number of differentiated neurons was decreased in human ES-NSPCs (0.52 ± 0.13 fold compared to control) as seen in mouse NSPCs (Additional file 6: Figure S5).

### CHD7 supports cell survival and proliferation of GICs

**Fig. 7:**
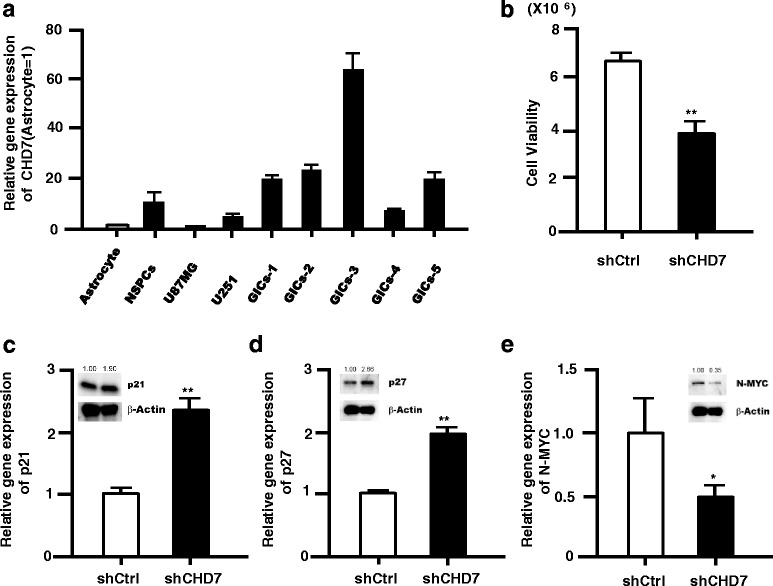
Functional analysis of CHD7 in glioma tumor initiating cells. **a**, Relative gene expression level of CHD7 was evaluated in GICs, NSPCs and glioma cell compared to astrocytes by qRT-PCR. **b**, CHD7 supports cell proliferation and/or survival in GICs (GICs-1). Lentiviral CHD7-shRNA expression significantly reduced cell proliferation and/or survival in GICs 5 days after infection. **c**–**e**, Gene expression of cell cycle arrest genes (**c**, p21; D, p27) was up-regulated and N-MYC was down-regulated (**e**) by lentiviral CHD7-shRNA gene silencing in GICs 5 days after infection. Error bars indicate S.D. values; *P < 0.05, **P < 0.01 versus control (*n* = 3); Student’s *t*-test. The protein level of p21 (**c**), p27 (**d**), and N-MYC (**e**) was quantified using Image J software, and relative protein levels (normalized by β-actin) are shown above the panel

GICs are known to maintain glioma, and the identification of key molecule(s) in the regulation of GICs may contribute to the development of novel glioma treatment strategies [9]. To test the function of CHD7 in human glioma, we firstly examined the CHD7 gene expression in normal astrocytes, NSPCs (human fetal brain derived), glioma cells (U87MG, U251), and GICs. Intriguingly, CHD7 was highly expressed in GICs (Fig. 7a). In silico analysis shows that CHD7 and CHD9 are well expressed in brain tumor, and CHD7 is expressed highly in Glioblastoma multiforme (GBM) compared to normal brain (Additional file 7: Figure S6). We examined the effect of CHD7 gene silencing in GICs, and found that lentiviral CHD7 gene silencing decreased the cell proliferation (0.56 ± 0.07 fold compared to control) (Fig. 7b). In addition, CHD7 gene silencing in GICs upregulated cell cycle arrest genes (p21 and p27) (Fig. 7c, d) and downregulated N-MYC, whose gene amplification was reported in GBM [26] (Fig. 7e).

## Discussion

In the present study, we identified Chd7, which is expressed in the fetal mouse brain, as a maintenance factor for NSPCs. Moreover, we showed that CHD7 gene expression is regulated by MIF in mouse NSPCs and human ES-NSPCs. In addition, we also showed that CHD7 supports the cell proliferation of GICs.

Some CHD family members have been reported to play a role in the neural development in mouse brain. Chd4 was required for Ezh2 mediated inhibition of gliogenesis in mouse embryonic brain [27]. In addition, Chd2 was required for embryonic neurogenesis regulating REST and also expressed in cultured neurospheres in mouse [28]. Depletion of Chd5 led to an inhibition of neurogenesis accompanied with the accumulation of undifferentiated cells in the mouse embryonic brain [29]. In adult mouse SVZ and hippocampus, the expression of Chd7 in actively dividing NSPCs was reported, and Chd7 contributed to the neurogenesis accompanied with the activation of Sox4 and Sox11 [16]. In the study, a slight increase in the number of Tbr2 cells in the SGZ of Chd7 mutant mouse (*Nestin*-CreER2:Chd7^fl/fl^) was reported [16]; however, the decrease of the Tbr2/KI67 double positive cells was observed in Chd7 mutant (Whi) mouse embryonic brain accompanied with the decreased neurogenesis observed in the present study. In addition, other groups also studied the *Nestin*-CreER2:Chd7^fl/fl^ mice and reported a decrease in number of KI67-positive cells in SVZ of embryonic brain [30]. Furthermore, another group showed that Chd7 maintained NSPCs quiescence status in mouse hippocampus using a different mouse model system (*GLAST*-CreER2:Chd7^fl/fl^) [17], showing Hes5 gene regulation by Chd7. We note that different phenotypes may occur depending on the mutant mouse type. However, in both of the different mouse models used here, Chd7 knockout led to a decrease of neurogenesis in the adult brain based on the NSPCs regulation, which is consistent with embryonic brain. In human CHARGE syndrome, the many different types of CHD7 mutations have been reported, and CHD7 protein expression in the lateral ventricle and hippocampus in human adult brain has been observed (http://www.proteinatlas.org). Thus, the analysis of the effects of CHD7 mutations in human brain on psychiatric disorders regulated by NSPCs may be important in the future.

We reported the TP53 regulation by MIF in a transcription independent manner in human glioma cells [9]. Intriguingly, MIF was expressed in nucleus and cytoplasmic fractions and antagonized TP53 intracellularly in the system studied. In addition, CHD7 could repress TP53 gene expression by the direct-bonding on the TP53 promoter, and the CHD7-TP53 signaling axis was reported in Chd7-null mouse neural crest cells, and fibroblasts from patients with CHARGE syndrome [31]. We examined the gene expression of TP53 in CHD7 gene silenced human ES-NSPCs and GICs; however, the TP53 gene was not upregulated in these systems (data not shown). In CHD5 study, CHD5 showed the dual roles of gene activation and repression in neurogenesis [29], showing the possibility CHD7 may have these dual roles depending on the context. A more precise analysis of TP53 regulation related to cell proliferation and apoptosis by CHD7 regulated by MIF in mouse and human NSPCs, GICs in both a transcription-dependent and -independent fashion may be of interest for future study.

In the present study, we observed the reduction of neurogenesis by Chd7 gene silencing in mouse NSPCs in vitro and the reduction of IPCs and Pax6/pH3 cells in Chd7 mutant embryonic brain in vivo. Using in vitro murine NSPC culture, we have shown that Chd7 regulates the gene expression of N-myc. N-myc conditional deletion reduced the IPC population in mouse developmental brain [32]. Thus, IPCs may be subject to regulation by the Chd7-N-myc axis in the embryonic mouse cortex. However, it has been reported that Chd7 cooperates with Sox10 to regulate the initiation of myelination and remyelination (genesis of differentiated OLs from OPCs, oligodendrocyte precursor cells) in vivo [18], suggesting possible diverse roles for Chd7 that vary in a manner dependent on spatial and developmental context. Indeed, Chd7 cooperates with PBAF in controlling the formation of neural crest cells based on the binding to the target gene’s enhancer region [13]. In addition, many binding partner proteins of CHD7 have been reported [20]. As a component of multimolecular complexes involving various binding partners, CHD7 may thus contribute to the determination of some cell lineages. High expression of Chd7 in neurons differentiated from mouse NSPCs compared to glial lineages cells was observed in vitro. Further study focused on epigenetic regulation on Chd7 may be also important to the understanding of this phenomenon. Moreover, we showed the CHD7 expression level in GICs compared to cultured primary astrocyte cells and neural stem/progenitor cells from human fetal brain in Fig. 7a, because astrocytes [33] and neural stem/progenitor cells [34, 35] may be the major cell origins of glioblastomas. Interestingly, mouse cortical neuron also generate glioma [36]. Thus, neural progenitor cells may be also　cellular source for GICs. Comparing CHD7 expression level and function in GICs, oligodendrocyte progenitors, and neural progenitor cells accompanied would be interesting for future study.

It is known that some CHD dysfunction occurs in cancer [37]. *In silico* analysis in the present showed the higher expression of CHD7 and CHD9 in brain tumor (Additional file 7: Figure S6). In addition, in TCGA database analysis, the overall survival of high-CHD7 expression patients tended to be shorter compared to that of low-CHD7 expression patients in GBM (data not shown), suggesting that CHD7 may support the proliferation of glioma cells and GICs. In contrast, CHD5 has been reported as a tumor suppressor gene in some tumors, such as in lung cancer [38]. In addition, intriguingly, low-expression of CHD7 was correlated to the survival of gemcitabine treated pancreatic ductal adenocarcinoma patients [39], suggesting the potential of CHD7 as a tumor suppresser gene like CHD5 in some tumors. Thus, functional analysis and identification of target genes and upstream regulators of CHD7 in CHD7-high expression tumors including liver and colorectal cancer may be of interest in this context.

We have reported the roles of MIF, which supports the cell proliferation and/or survival in mouse NSPCs and GICs. In mouse NSPCs, MIF regulates many signaling molecules, including Erk, Stat3, AMPK, and Sox6 [7, 8]. In the developmental mouse brain, the expression pattern of Sox6 and Chd7 differ, although both genes are regulated by MIF in NSPCs in vitro. However, SOX6 gene transcript was downregulated by CHD7 gene silencing in human ES-NSPCs in vitro (data not shown). Thus, we are currently analyzing the detailed signaling mechanism regulated by MIF in NSPCs and GICs including the regulation of both miR and lncRNA in addition to SOX6. Detailed analyses of the epigenetic regulation by MIF-CHD7 pathway in NSPCs may help to clarify MIF function in these cells, and contribute to the development of new applications for MIF biology in regenerative medicine and induced pluripotent stem cells (iPSCs).

In this study, we focused on CHD7 function in NSPCs derived from mouse embryonic brains, human ES-NSPCs, and GICs in vitro, showing the MIF function as a cell proliferation factor. Importantly, CHD7 is regulated by MIF in mouse NSPCs and human ES-NSPCs. Investigations of CHD7 function in gliogenesis at different developmental stages and in other neural diseases, such as stroke and spinal cord injury, that are associated with regenerative neurogenesis remain for future study. It will also be useful to conduct analyses of the regulation of CHD7 binding to the histone (H3H4me1) and itsenhancer(s) in NSPCs. Finally, functional analyses of CHD7 especially in human gliomas and GICs, may help pave the way for evaluating CHD7 as a therapeutic target.

## Additional files

Additional file 1:
**Table S1.** Primer sequence. (DOCX 14 kb)
Additional file 2:
**Figure S1.** Chd7 gene knockdown decreases NSPCs proliferation. A, Chd7 targeting using lentiviral shRNA significantly reduced mouse NSPC growth compared to control shRNA, as assessed using a Cell Titer-Glo Assay Kit 4 days after infection. B, The gene expression level of Chd7 was quantified by qRT-PCR in NSPCs 4 days after infection. Error bars indicate S.D. values; **P* < 0.05, ***P* < 0.01 versus control; Student’s *t*-test from three independent experiments. C, We counted the number of Chd7-positive cells in each of the differentiated neural cells derived from embryonic day 14.5 brain GE-NSPCs after 5 days of differentiation without growth factors. Error bars indicate S.D. values and value shows the average of three independent experiments. D, Chd7 protein expression was reduced in the differentiated neuronal cells in the absence of growth factors derived from embryonic day 14.5 brain GE-NSPCs 2 days after differentiation. The level of Chd7 was quantified using Image J software (NIH, Bethesda, MD) and relative Chd7 protein levels (normalized by β-actin) are shown below the panel. (PPT 278 kb)
Additional file 3:
**Figure S2.** MIF regulated Chd7 can support cell survival and/or proliferative ability in NSPCs. A, Changes in cell proliferation with or without MIF treatment (400 ng/ml) in mouse NSPCs were observed using a CellTiter Glo Luminescent Cell kit. The increase in cell viability by MIF treatment was inhibited by lentiviral Chd7 gene knockdown 4 days after infection (*n* = 3). Error bars indicate S.D. values; **P* < 0.05, ***P* < 0.01 versus control; Student’s *t*-test from three independent experiments. B. Neurospheres infected with lentivirus expressing either control or Chd7-targeting shRNA were cultured in the presence of both EGF and FGF2 for 5 days, and then dissociated and cultured onto the poly-D-lysine coated glass slip for 5DIV in the absence of growth factors. The expression of neural marker (βIII-tubulin), an astrocyte marker (GFAP), or an oligodendrocyte marker (CNPase) was evaluated by immunocytochemistry. Error bars indicate S.D. values and data was derived at least three independent experiments. (PPT 224 kb)
Additional file 4:
**Figure S3.** Analysis of mitotic cells in the apical ventricular zone and matured neural cells in the cortical plate in Chd7 mutant mouse. A, Cells immunopositive for phosphorylated histone H3 staining (pH3, green signal) indicate cells in mitosis. The number of pH3/Pax6 (red)-double positive cells in Chd7 mutant mouse (Chd7^Whi/+^, *n* = 8) was counted compared to the wild-type littermates (*n* = 6) at embryonic day 14.5 brain. B, Cells that are immunopositive for NeuN in the cortical plate (CP) were counted at embryonic day 14.5 brain (wild-type littermates, *n* = 4; Chd7^Whi/+^, *n* = 4). Error bars indicate S.D. values; **P* < 0.05 versus control; Student’s *t*-test. Scale bar; 20 μm.(A), 50 μm (B). (PPT 1259 kb)
Additional file 5:
**Figure S4.** Identification of MIF regulated genes in human ES-NSPCs. A-D, Change of targets and MIF gene expression level by lentiviral MIF gene knockdown were quantified in human ES- NSPCs 2 days after infection. Error bars indicate S.D. values; **P* < 0.05, ***P* < 0.01 versus control; Student’s *t*-test from three independent experiments. (PPT 198 kb)
Additional file 6:
**Figure S5.** Differentiation potential of human ES- NSPCs derived fromCHD7 knockdown. A, Human ES-NSPCs were infected with lentivirus expressing either CHD7 shRNA or control shRNA for 2 days and then differentiated in each differentiation medium for 2 weeks (neuron) or 3 weeks (astrocytes). Representative images of differentiated cells, which were fixed and subjected to immunocytochemical analyses labeled with a neuronal marker (βIII-tubulin) or astrocyte marker (GFAP), were shown. Scale bar: 20 μm. B, βIII-tubulin 1and GFAP positive number in DAPI-positive cells was counted, and the data were expresses as percentages. (PPT 1018 kb)
Additional file 7:
**Figure S6.** *In silico* analysis of CHD family expression in GBM. A, Relative CHD family mRNA expression data in different human cancers were obtained from the public version of the Oncomine (www.oncomine.org). B, CHD7 gene expression of brain tumors compared to normal brain was analyzed *in silico* using the REMBRANDT data base (http://rembrandt.nci.nih.gov). (PPT 332 kb)

## Abbreviations

ES: Embryonic stem
GBM: Glioblastoma multiforme
GE: Ganglionic eminence
GICs: Glioma initiating cells
iPCs: Intermediate progenitor cells
iPSCs: Induced pluripotent stem cells
NSPCs: Neural stem progenitor cells
SVZ: Subventricular zone
