# Supplementary material for: CHD7 promotes proliferation of neural stem cells mediated by MIF
Source: Mol Brain. 2016 Dec 13;9:96. doi: 10.1186/s13041-016-0275-6 (PMC5154087; doi:10.1186/s13041-016-0275-6)
Supplement: Additional file 1: — Table S1. Primer sequence. (DOCX 14 kb) [file 13041_2016_275_MOESM1_ESM.docx]

**Table S1 Primer sequence**

SYBAR probe

| **Target** | **Primer name** | **Sequence** |
| --- | --- | --- |
| Chd7 | Chd7 Fwd  Chd7 Rev | GGAGAACCCTGAGTTTGCTG CCCTGAAGTAGAGGCGACAG |
| Pax6 | Pax6 Fwd  Pax6 Rev | CGGAGGGAGTAAGCCAAGAG  TCTGTCTCGGATTTCCCAAG |
| N-Myc | N-Myc Fwd  N-Myc Rev | GTGTCTGTTCCAGCTACTGC CATCTTCCTCCTCGTCATC |

TaqMan probe (Thermo Fisher Scientific)

| **Target** | **Probe ID** |
| --- | --- |
| CHD7 | Hs00215010 |
| PAX6 | Hs00240871 |
| NMYC | Hs 00232074 |
| MIF | Hs 00236988 |
| HES5 | Hs01387463 |
| p21 | Hs00355782 |
| p27 | Hs0015277 |
