# Supplementary material for: CHD7 promotes proliferation of neural stem cells mediated by MIF
Source: Mol Brain. 2016 Dec 13;9:96. doi: 10.1186/s13041-016-0275-6 (PMC5154087; doi:10.1186/s13041-016-0275-6)
Supplement: Additional file 2: — Figure S1. Chd7 gene knockdown decreases NSPCs proliferation. A, Chd7 targeting using lentiviral shRNA significantly reduced mouse NSPC growth compared to control shRNA, as assessed using a Cell Titer-Glo Assay Kit 4 days after infection. B, The gene expression level of Chd7 was quantified by qRT-PCR in NSPCs 4 days after infection. Error bars indicate S.D. values; *P < 0.05, **P < 0.01 versus control; Student’s t-test from three independent experiments. C, We counted the number of Chd7-positive cells in each of the differentiated neural cells derived from embryonic day 14.5 brain GE-NSPCs after 5 days of differentiation without growth factors. Error bars indicate S.D. values and value shows the average of three independent experiments. D, Chd7 protein expression was reduced in the differentiated neuronal cells in the absence of growth factors derived from embryonic day 14.5 brain GE-NSPCs 2 days after differentiation. The level of Chd7 was quantified using Image J software (NIH, Bethesda, MD) and relative Chd7 protein levels (normalized by β-actin) are shown below the panel. (PPT 278 kb) [file 13041_2016_275_MOESM2_ESM.ppt]

## Slide 1
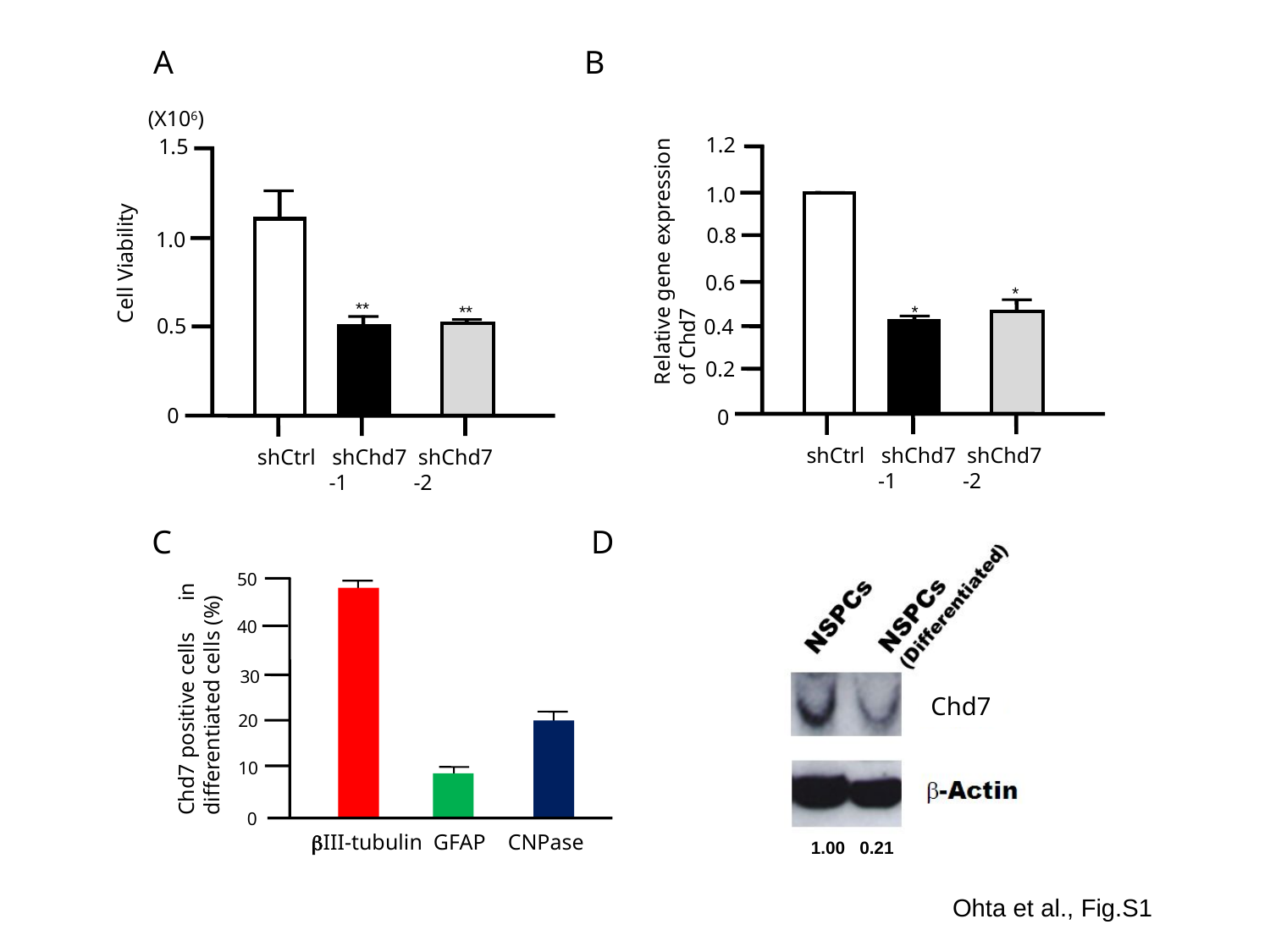

A 　　 B
(X106)
1.2
1.5
1.0
0.8
1.0
Relative gene expression
of Chd7
Cell Viability
0.6
*
**
*
**
0.5
0.4
0.2
0
0
shCtrl shChd7 shChd7
 -1 -2
shCtrl shChd7 shChd7
 -1 -2
C 　 　D
50
40
30
Chd7 positive cells　in
differentiated cells (%)
Chd7
20
10
0
βIII-tubulin GFAP CNPase
1.00 0.21
Ohta et al., Fig.S1
