# Supplementary material for: CHD7 promotes proliferation of neural stem cells mediated by MIF
Source: Mol Brain. 2016 Dec 13;9:96. doi: 10.1186/s13041-016-0275-6 (PMC5154087; doi:10.1186/s13041-016-0275-6)
Supplement: Additional file 3: — Figure S2. MIF regulated Chd7 can support cell survival and/or proliferative ability in NSPCs. A, Changes in cell proliferation with or without MIF treatment (400 ng/ml) in mouse NSPCs were observed using a CellTiter Glo Luminescent Cell kit. The increase in cell viability by MIF treatment was inhibited by lentiviral Chd7 gene knockdown 4 days after infection (n = 3). Error bars indicate S.D. values; *P < 0.05, **P < 0.01 versus control; Student’s t-test from three independent experiments. B. Neurospheres infected with lentivirus expressing either control or Chd7-targeting shRNA were cultured in the presence of both EGF and FGF2 for 5 days, and then dissociated and cultured onto the poly-D-lysine coated glass slip for 5DIV in the absence of growth factors. The expression of neural marker (βIII-tubulin), an astrocyte marker (GFAP), or an oligodendrocyte marker (CNPase) was evaluated by immunocytochemistry. Error bars indicate S.D. values and data was derived at least three independent experiments. (PPT 224 kb) [file 13041_2016_275_MOESM3_ESM.ppt]

## Slide 1
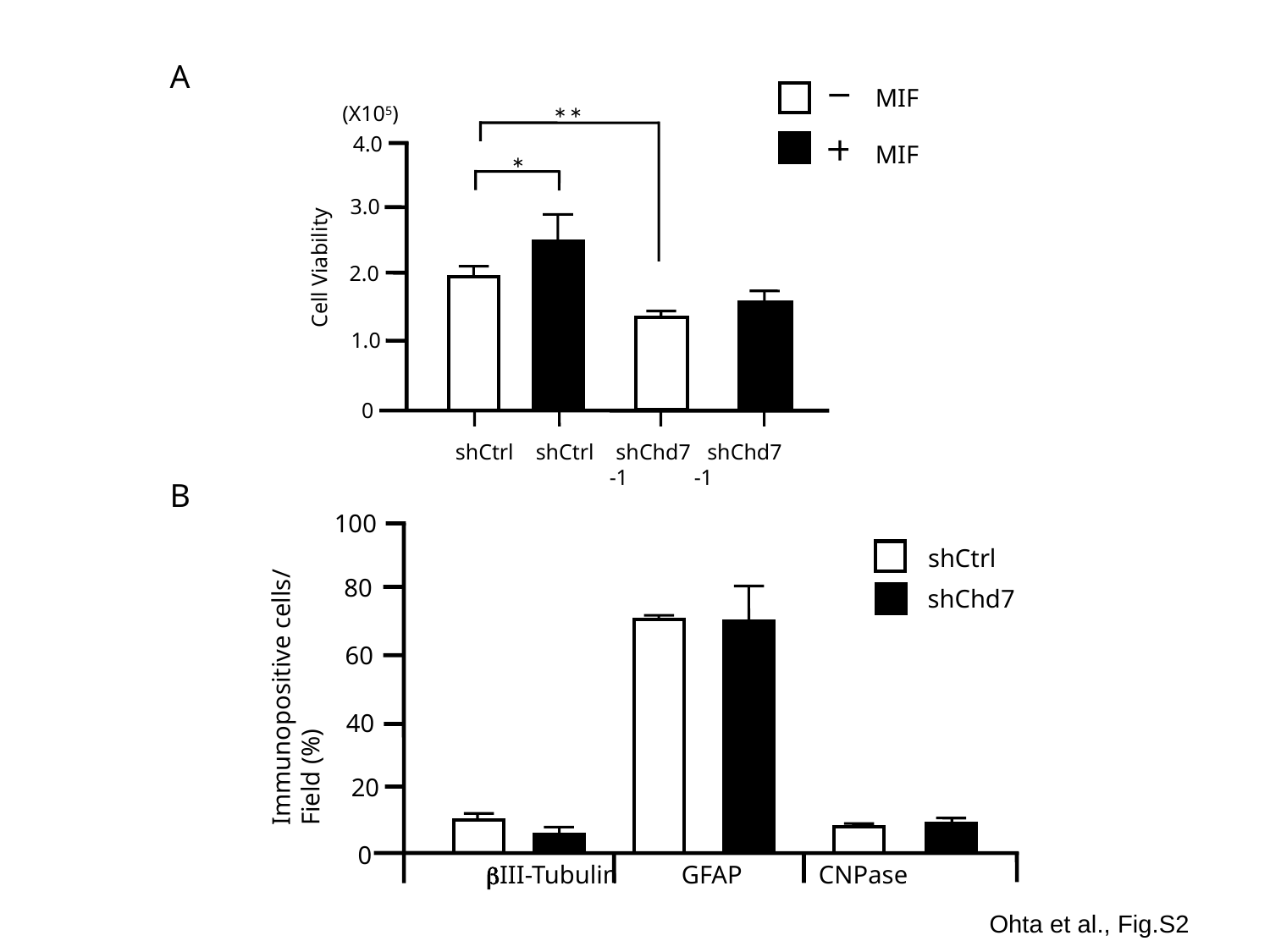

A
B
MIF
**
(X105)
4.0
MIF
*
3.0
Cell Viability
2.0
1.0
0
shCtrl shCtrl shChd7 shChd7
 -1 -1
100
shCtrl
80
shChd7
60
Immunopositive cells/
Field (%)
40
20
0
βIII-Tubulin GFAP CNPase
Ohta et al., Fig.S2
