# Supplementary material for: CHD7 promotes proliferation of neural stem cells mediated by MIF
Source: Mol Brain. 2016 Dec 13;9:96. doi: 10.1186/s13041-016-0275-6 (PMC5154087; doi:10.1186/s13041-016-0275-6)
Supplement: Additional file 4: — Figure S3. Analysis of mitotic cells in the apical ventricular zone and matured neural cells in the cortical plate in Chd7 mutant mouse. A, Cells immunopositive for phosphorylated histone H3 staining (pH3, green signal) indicate cells in mitosis. The number of pH3/Pax6 (red)-double positive cells in Chd7 mutant mouse (Chd7Whi/+, n = 8) was counted compared to the wild-type littermates (n = 6) at embryonic day 14.5 brain. B, Cells that are immunopositive for NeuN in the cortical plate (CP) were counted at embryonic day 14.5 brain (wild-type littermates, n = 4; Chd7Whi/+, n = 4). Error bars indicate S.D. values; *P < 0.05 versus control; Student’s t-test. Scale bar; 20 μm.(A), 50 μm (B). (PPT 1259 kb) [file 13041_2016_275_MOESM4_ESM.ppt]

## Slide 1
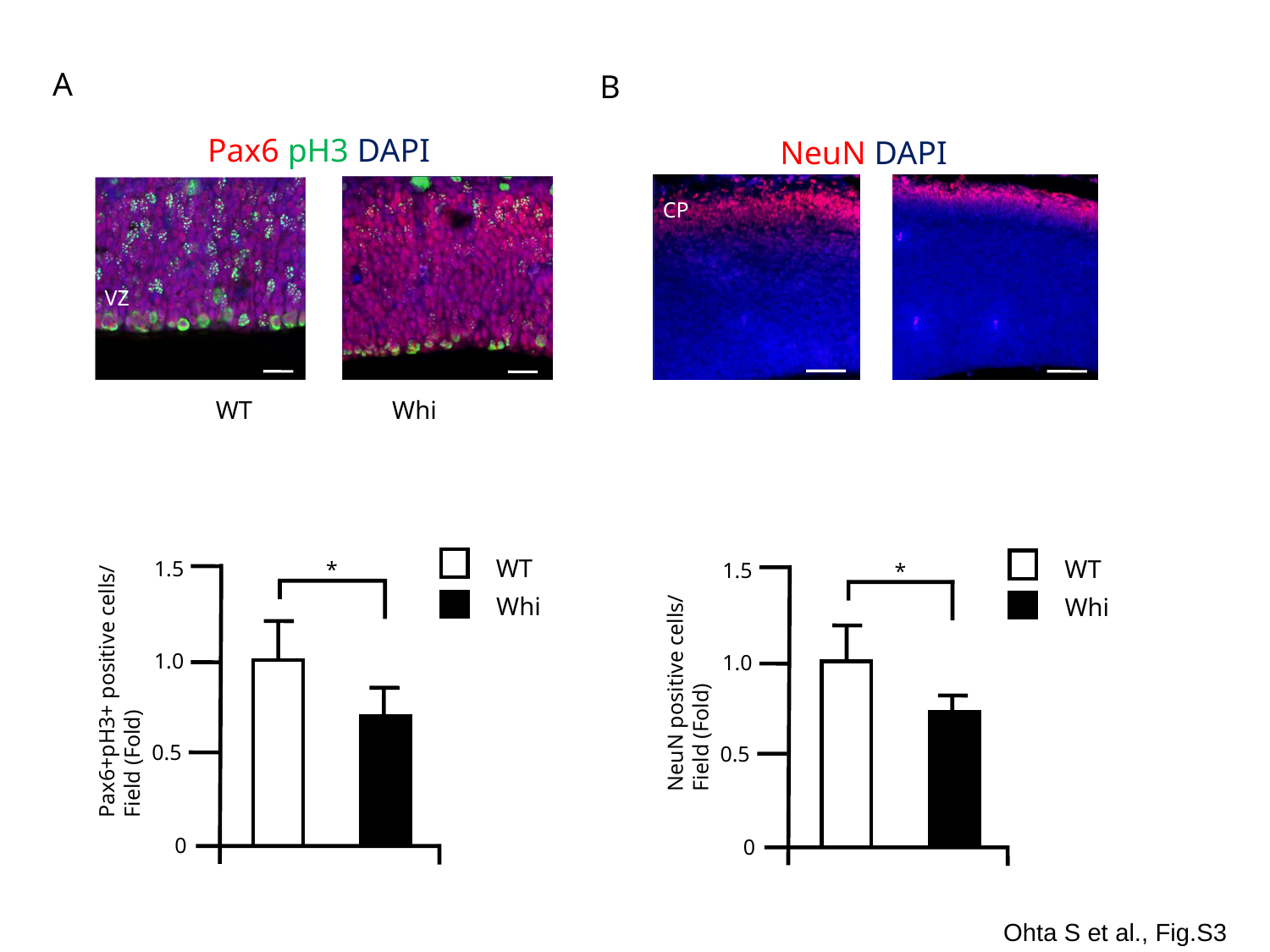

A
B
Pax6 pH3 DAPI
NeuN DAPI
CP
VZ
WT Whi
WT
WT
1.5
*
1.5
*
Whi
Whi
1.0
1.0
Pax6+pH3+ positive cells/
Field (Fold)
NeuN positive cells/
Field (Fold)
0.5
0.5
0
0
Ohta S et al., Fig.S3
