# Supplementary material for: CHD7 promotes proliferation of neural stem cells mediated by MIF
Source: Mol Brain. 2016 Dec 13;9:96. doi: 10.1186/s13041-016-0275-6 (PMC5154087; doi:10.1186/s13041-016-0275-6)
Supplement: Additional file 5: — Figure S4. Identification of MIF regulated genes in human ES-NSPCs. A-D, Change of targets and MIF gene expression level by lentiviral MIF gene knockdown were quantified in human ES- NSPCs 2 days after infection. Error bars indicate S.D. values; *P < 0.05, **P < 0.01 versus control; Student’s t-test from three independent experiments. (PPT 198 kb) [file 13041_2016_275_MOESM5_ESM.ppt]

## Slide 1
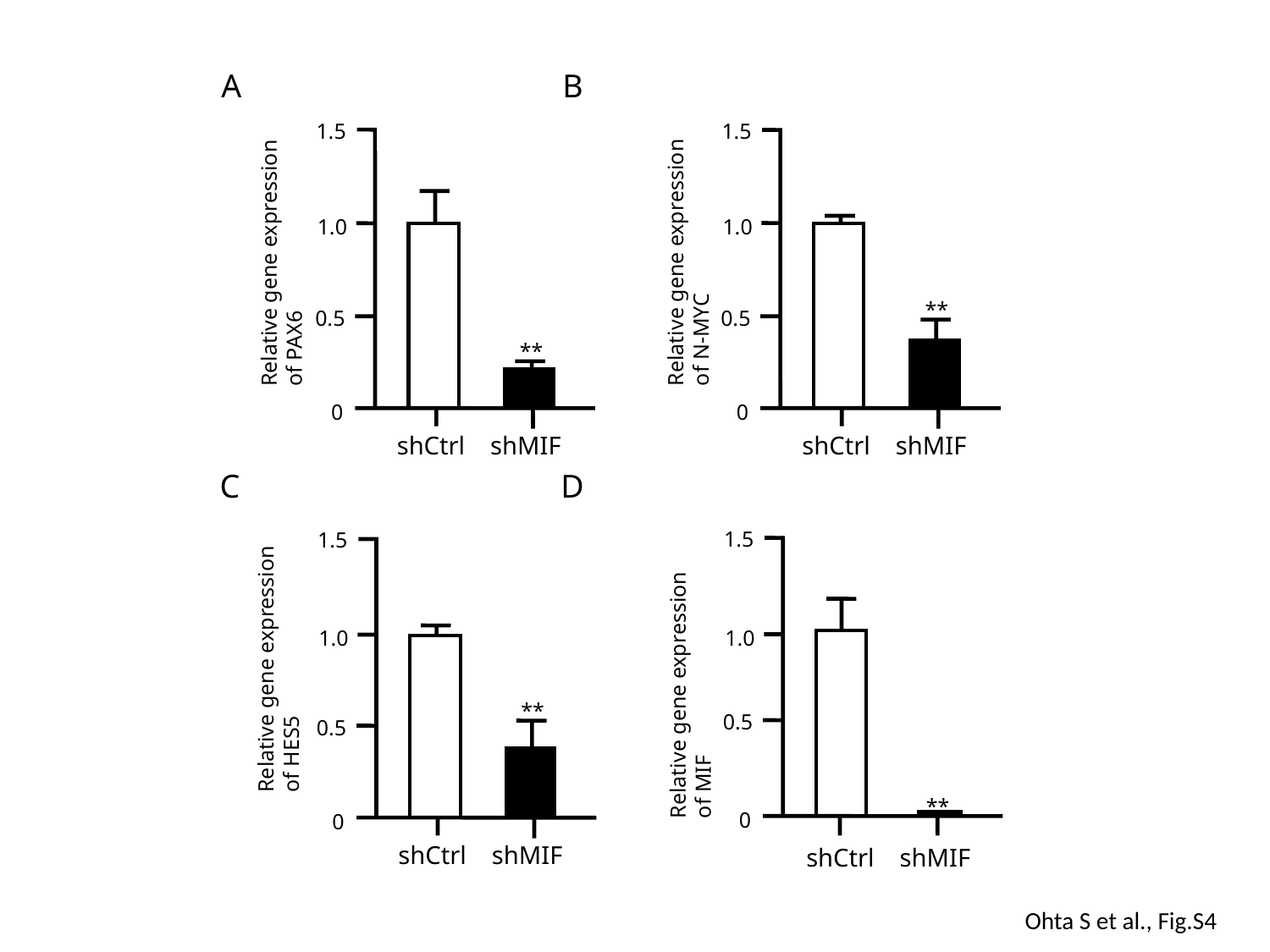

A B
1.5
1.5
1.0
1.0
Relative gene expression
of N-MYC
Relative gene expression
of PAX6
**
0.5
0.5
**
0
0
shCtrl shMIF
shCtrl shMIF
C D
1.5
1.5
Relative gene expression
of MIF
1.0
1.0
Relative gene expression
of HES5
**
0.5
0.5
**
0
0
shCtrl shMIF
shCtrl shMIF
Ohta S et al., Fig.S4
