# Supplementary material for: CHD7 promotes proliferation of neural stem cells mediated by MIF
Source: Mol Brain. 2016 Dec 13;9:96. doi: 10.1186/s13041-016-0275-6 (PMC5154087; doi:10.1186/s13041-016-0275-6)
Supplement: Additional file 6: — Figure S5. Differentiation potential of human ES- NSPCs derived fromCHD7 knockdown. A, Human ES-NSPCs were infected with lentivirus expressing either CHD7 shRNA or control shRNA for 2 days and then differentiated in each differentiation medium for 2 weeks (neuron) or 3 weeks (astrocytes). Representative images of differentiated cells, which were fixed and subjected to immunocytochemical analyses labeled with a neuronal marker (βIII-tubulin) or astrocyte marker (GFAP), were shown. Scale bar: 20 μm. B, βIII-tubulin 1and GFAP positive number in DAPI-positive cells was counted, and the data were expresses as percentages. (PPT 1018 kb) [file 13041_2016_275_MOESM6_ESM.ppt]

## Slide 1
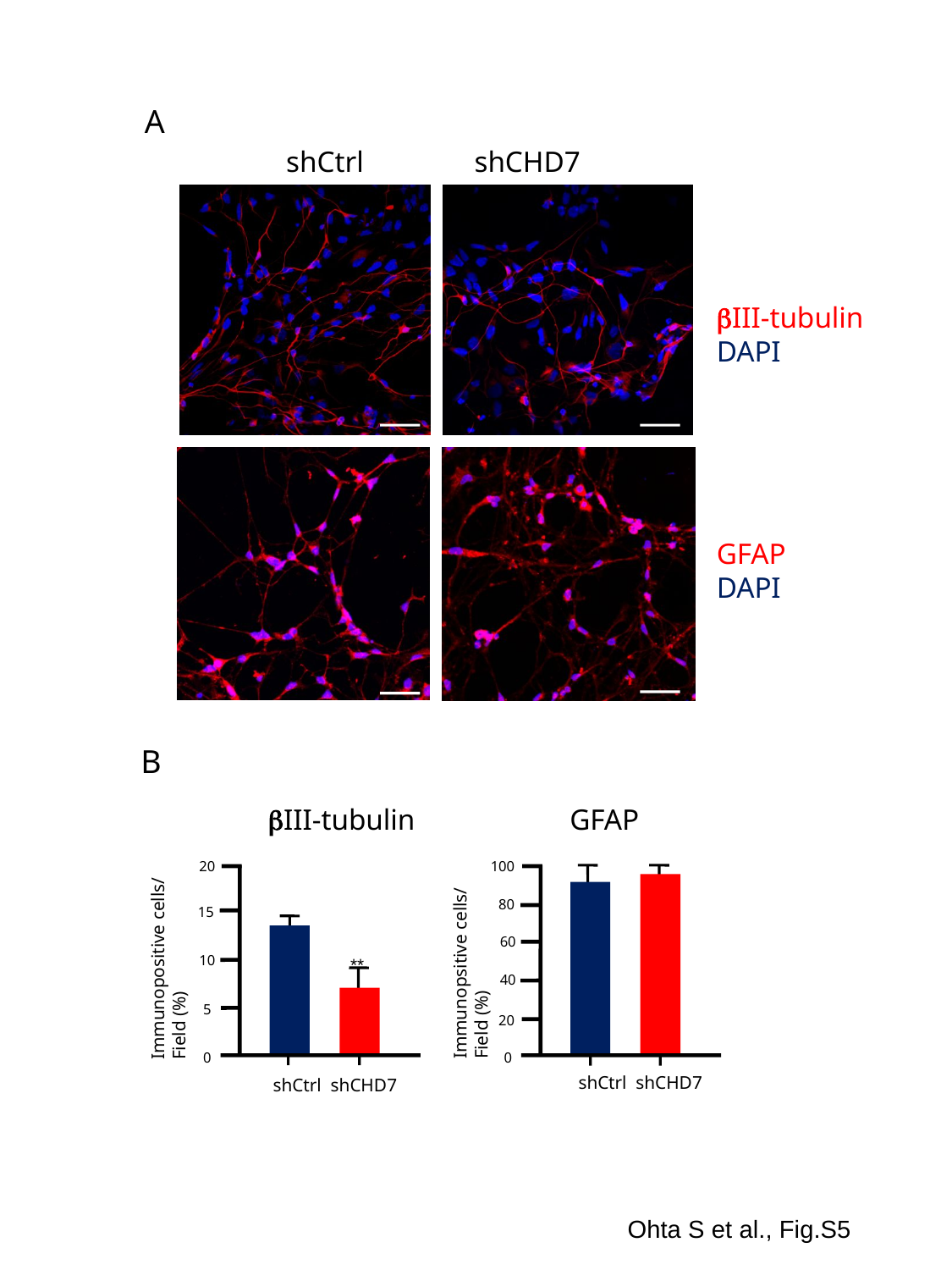

A
shCtrl shCHD7
βIII-tubulin
DAPI
GFAP
DAPI
B
Immunopsitive cells/
Field (%)
βIII-tubulin
GFAP
Immunopositive cells/
Field (%)
20
100
80
15
60
10
**
40
5
20
0
0
shCtrl shCHD7
shCtrl shCHD7
Ohta S et al., Fig.S5
