# Supplementary figures and images for: CHD7 promotes proliferation of neural stem cells mediated by MIF
Source: Mol Brain. 2016 Dec 13;9:96. doi: 10.1186/s13041-016-0275-6 (PMC5154087; doi:10.1186/s13041-016-0275-6)

## Slide 1
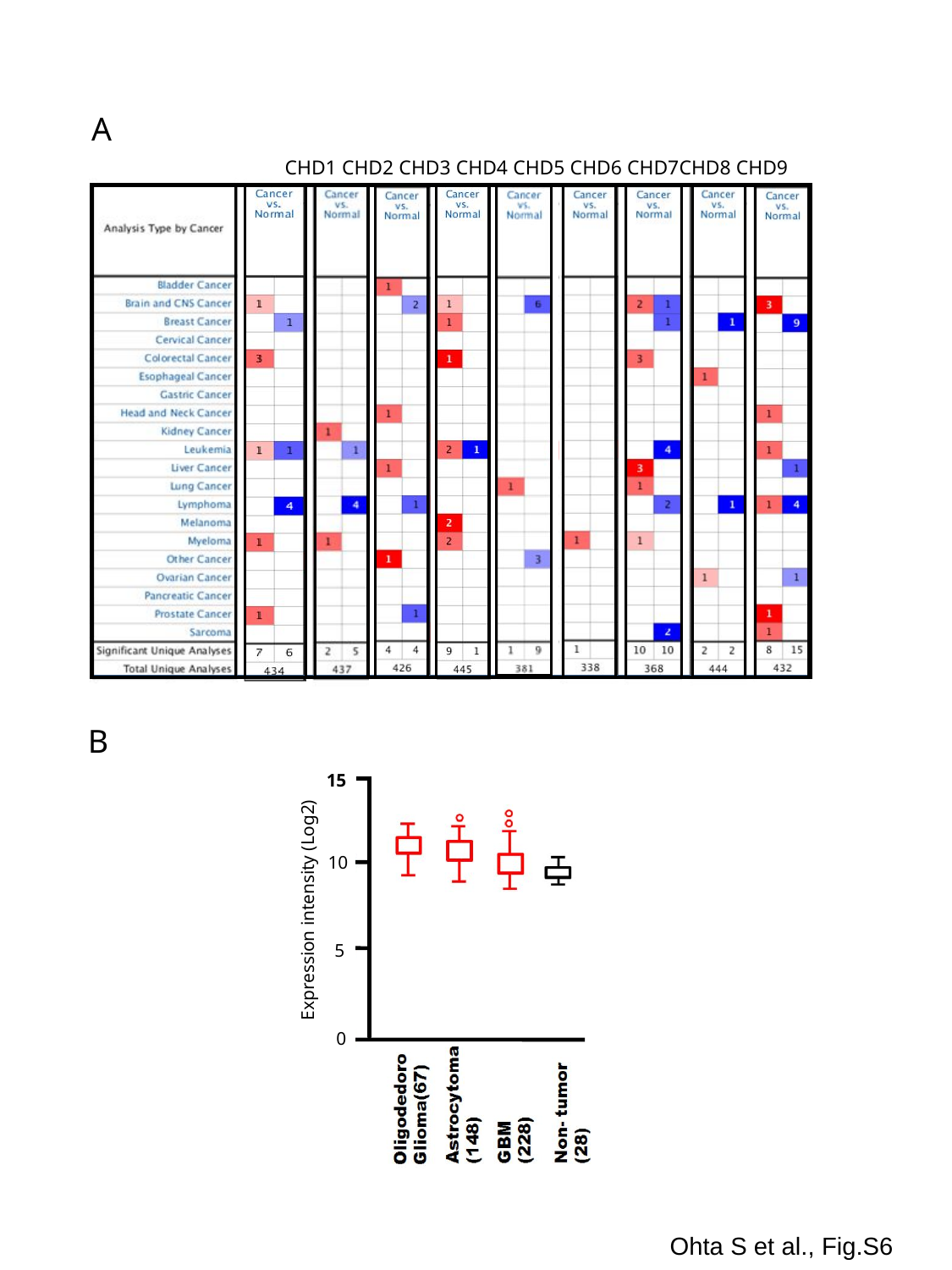

A
CHD1 CHD2 CHD3 CHD4 CHD5 CHD6 CHD7CHD8 CHD9
B
15
10
Expression intensity (Log2)
5
0
Ohta S et al., Fig.S6

Supplement: Additional file 7: — Figure S6. In silico analysis of CHD family expression in GBM. A, Relative CHD family mRNA expression data in different human cancers were obtained from the public version of the Oncomine (www.oncomine.org). B, CHD7 gene expression of brain tumors compared to normal brain was analyzed in silico using the REMBRANDT data base (http://rembrandt.nci.nih.gov). (PPT 332 kb) [file 13041_2016_275_MOESM7_ESM.ppt]
